# 2-(4-Bromo-1*H*-indol-3-yl)acetonitrile

**DOI:** 10.1107/S1600536811054936

**Published:** 2012-01-18

**Authors:** Qiu-Xia Mao, Chen-Guang Zhang, Jin-Feng Li

**Affiliations:** a College of Chemistry and Chemical, Engineering, Southeast UniVersity, Nanjing 211189, People’s Republic of China

## Abstract

In the title compound, C_10_H_7_BrN_2_, the non-H atoms, except the N atom of the acetonitrile group and the C atom bonded to it, lie in the least-squares plane defined by the atoms of the indole ring system (r.m.s deviation = 0.019 Å), with the N and C atom of the cyano group displaced by 2.278 (1) and 1.289 (1) Å, respectively, out of that plane. In the crystal, N—H⋯N hydrogen bonds link the mol­ecules into a *C*(7) chain along [100].

## Related literature

For natural products with a bromo indole moiety, see: Walker *et al.* (2009 ▶). For the use of 4-bromo indole derivatives in the synthesis of biologically active compounds, see: Hendrickson & Wang (2004 ▶); Giraud *et al.* (2011 ▶). For the structures of related halo indoles, see: Kunzer & Wendt (2011 ▶).
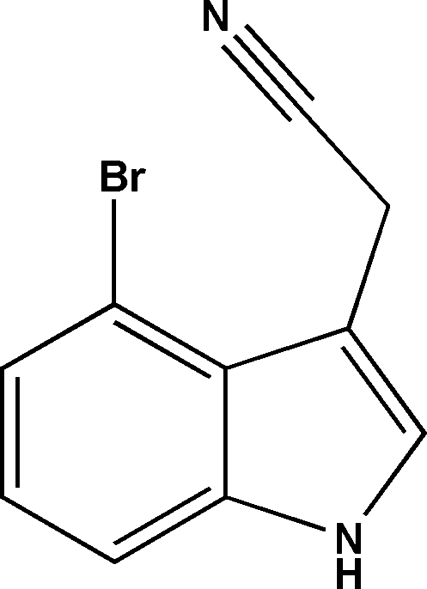


## Experimental

### 

#### Crystal data

C_10_H_7_BrN_2_
*M* *_r_* = 235.09
Monoclinic, 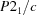

*a* = 8.3971 (17) Å
*b* = 11.237 (2) Å
*c* = 9.979 (2) Å
β = 104.82 (3)°
*V* = 910.2 (3) Å^3^
*Z* = 4
Mo *K*α radiation
μ = 4.46 mm^−1^
*T* = 293 K
0.20 × 0.20 × 0.20 mm

#### Data collection

Rigaku SCXmini diffractometer
Absorption correction: multi-scan (*CrystalClear*; Rigaku, 2005 ▶) *T*_min_ = 0.983, *T*_max_ = 0.983
9047 measured reflections
2082 independent reflections
1489 reflections with *I* > 2σ(*I*)
*R*_int_ = 0.115

#### Refinement

*R*[*F* ^2^ > 2σ(*F* ^2^)] = 0.073
*wR*(*F* ^2^) = 0.188
*S* = 1.09
2082 reflections
118 parameters
H-atom parameters constrained
Δρ_max_ = 0.64 e Å^−3^
Δρ_min_ = −1.84 e Å^−3^

### 

Data collection: *CrystalClear* (Rigaku, 2005 ▶); cell refinement: *CrystalClear*; data reduction: *CrystalClear*; program(s) used to solve structure: *SHELXS97* (Sheldrick, 2008 ▶); program(s) used to refine structure: *SHELXL97* (Sheldrick, 2008 ▶); molecular graphics: *DIAMOND* (Brandenburg & Putz, 2005 ▶); software used to prepare material for publication: *SHELXL97*.

## Supplementary Material

Crystal structure: contains datablock(s) I, global. DOI: 10.1107/S1600536811054936/lr2041sup1.cif

Structure factors: contains datablock(s) I. DOI: 10.1107/S1600536811054936/lr2041Isup2.hkl

Supplementary material file. DOI: 10.1107/S1600536811054936/lr2041Isup3.cml

Additional supplementary materials:  crystallographic information; 3D view; checkCIF report

## Figures and Tables

**Table 1:** Hydrogen-bond geometry (Å, °)

*D*—H⋯*A*	*D*—H	H⋯*A*	*D*⋯*A*	*D*—H⋯*A*
N1—H1*A*⋯N2^i^	0.86	2.45	3.218 (7)	148

Symmetry code: (i) 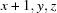.

**Fig. 1.**
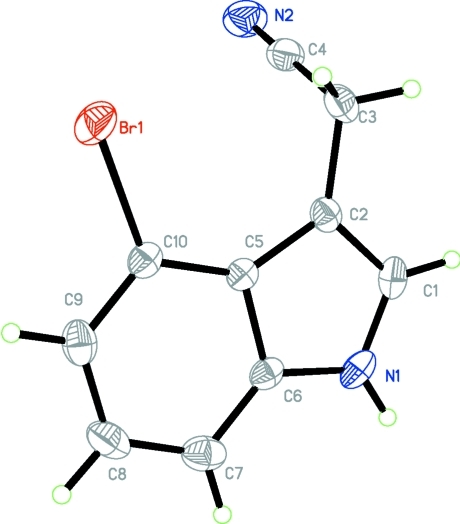
The molecular structure of the title compound. Displacement ellipsoids are drawn at the 30% probability level.

**Fig. 2.**
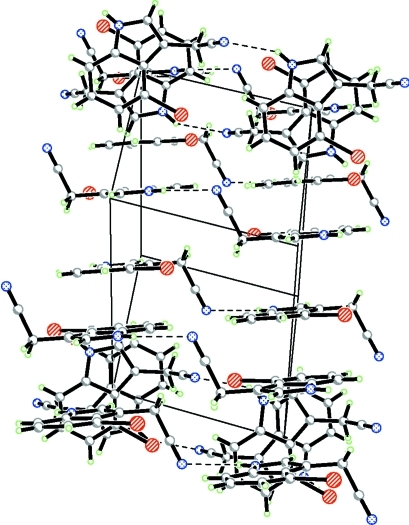
A packing view. Intermolecular hydrogen bonds are shown as dashed lines.

## supplementary crystallographic information

### Comment

The derivatives of halo indole present in several natural products (Walker *et al.*, 2009) are also excellent intermediates for the synthesis of many biological active compounds (Giraud *et al.*, 2011; Hendrickson & Wang, 2004) . As part of our interest in these materials, we report the crystal structure of the title compound.

The molecular structure of the title compound is shown in Fig. 1. The non-H atoms, except the nitrogen of the acetonitrile moiety and the carbon atom bonded to it, are lying in the least-squares plane defined by the atoms of the indole ring system (r.m.s deviation= 0.019 Å ), with the nitrogen and carbon of the cyano moiety shifted by 2.278 (1) and 1.289 (1) Å, respectively, out of that plane.

In the crystal, N1—H1A···N2 hydrogen bonds link the molecules into chain along the [100] direction (Fig. 2).

### Experimental

The title compound was obtained commercially from ChemFuture PharmaTech, Ltd (Nanjing, Jiangsu). Crystals suitable for X-ray diffraction were obtained by slow evaporation from a methanol solution.

### Refinement

All H atoms attached to C atoms and N atoms were fixed geometrically and treated as riding with C—H = 0.93 Å (CH), C—H = 0.97 Å (CH_2_), and N—H = 0.86 Å with *U*_iso_(H) = 1.2*U*_eq_.

### Crystal data

**Table:**

C_10_H_7_BrN_2_	*F*(000) = 464
*M**_r_* = 235.09	*D*_x_ = 1.715 Mg m^−^^3^
Monoclinic, *P*2_1_/*c*	Mo *K*α radiation, λ = 0.71073 Å
Hall symbol: -P 2ybc	Cell parameters from 2082 reflections
*a* = 8.3971 (17) Å	θ = 3.1–27.5°
*b* = 11.237 (2) Å	µ = 4.46 mm^−^^1^
*c* = 9.979 (2) Å	*T* = 293 K
β = 104.82 (3)°	Prism, colourless
*V* = 910.2 (3) Å^3^	0.20 × 0.20 × 0.20 mm
*Z* = 4	

### Data collection

**Table:**

Rigaku SCXmini diffractometer	2082 independent reflections
Radiation source: fine-focus sealed tube	1489 reflections with *I* > 2σ(*I*)
graphite	*R*_int_ = 0.115
Detector resolution: 13.6612 pixels mm^-1^	θ_max_ = 27.5°, θ_min_ = 3.1°
CCD_Profile_fitting scans	*h* = −10→10
Absorption correction: multi-scan (*CrystalClear*; Rigaku, 2005)	*k* = −14→14
*T*_min_ = 0.983, *T*_max_ = 0.983	*l* = −12→12
9047 measured reflections	

### Refinement

**Table:**

Refinement on *F*^2^	Primary atom site location: structure-invariant direct methods
Least-squares matrix: full	Secondary atom site location: difference Fourier map
*R*[*F*^2^ > 2σ(*F*^2^)] = 0.073	Hydrogen site location: inferred from neighbouring sites
*wR*(*F*^2^) = 0.188	H-atom parameters constrained
*S* = 1.09	*w* = 1/[σ^2^(*F*_o_^2^) + (0.0915*P*)^2^] where *P* = (*F*_o_^2^ + 2*F*_c_^2^)/3
2082 reflections	(Δ/σ)_max_ < 0.001
118 parameters	Δρ_max_ = 0.64 e Å^−^^3^
0 restraints	Δρ_min_ = −1.84 e Å^−^^3^

### Special details

**Table:**

Geometry. All e.s.d.'s (except the e.s.d. in the dihedral angle between two l.s. planes) are estimated using the full covariance matrix. The cell e.s.d.'s are taken into account individually in the estimation of e.s.d.'s in distances, angles and torsion angles; correlations between e.s.d.'s in cell parameters are only used when they are defined by crystal symmetry. An approximate (isotropic) treatment of cell e.s.d.'s is used for estimating e.s.d.'s involving l.s. planes.
Refinement. Refinement of *F*^2^ against ALL reflections. The weighted *R*-factor *wR* and goodness of fit *S* are based on *F*^2^, conventional *R*-factors *R* are based on *F*, with *F* set to zero for negative *F*^2^. The threshold expression of *F*^2^ > σ(*F*^2^) is used only for calculating *R*-factors(gt) *etc*. and is not relevant to the choice of reflections for refinement. *R*-factors based on *F*^2^ are statistically about twice as large as those based on *F*, and *R*- factors based on ALL data will be even larger.

### Fractional atomic coordinates and isotropic or equivalent isotropic displacement parameters (Å^2^)

**Table:**

	*x*	*y*	*z*	*U*_iso_*/*U*_eq_	
Br1	0.72608 (9)	1.02919 (6)	0.23625 (7)	0.0565 (3)	
C10	0.9402 (7)	0.9743 (4)	0.2351 (5)	0.0344 (12)	
N1	1.1261 (6)	0.7932 (4)	0.0298 (5)	0.0433 (11)	
H1A	1.2109	0.7600	0.0124	0.052*	
C5	0.9637 (6)	0.8989 (4)	0.1306 (5)	0.0285 (10)	
C2	0.8646 (6)	0.8483 (4)	0.0068 (5)	0.0341 (11)	
C9	1.0709 (8)	1.0096 (4)	0.3397 (6)	0.0447 (14)	
H9A	1.0531	1.0623	0.4063	0.054*	
C6	1.1290 (6)	0.8616 (4)	0.1422 (5)	0.0333 (11)	
C8	1.2297 (8)	0.9690 (5)	0.3493 (7)	0.0520 (15)	
H8A	1.3158	0.9924	0.4234	0.062*	
C1	0.9703 (8)	0.7850 (5)	−0.0511 (6)	0.0410 (13)	
H1B	0.9393	0.7426	−0.1338	0.049*	
C3	0.6815 (6)	0.8582 (5)	−0.0559 (5)	0.0439 (13)	
H3A	0.6489	0.9410	−0.0547	0.053*	
H3B	0.6565	0.8326	−0.1519	0.053*	
C4	0.5863 (7)	0.7872 (5)	0.0176 (6)	0.0444 (13)	
N2	0.5110 (7)	0.7337 (5)	0.0738 (6)	0.0608 (14)	
C7	1.2599 (7)	0.8940 (6)	0.2491 (5)	0.0490 (15)	
H7A	1.3656	0.8664	0.2540	0.059*	

### Atomic displacement parameters (Å^2^)

**Table:**

	*U*^11^	*U*^22^	*U*^33^	*U*^12^	*U*^13^	*U*^23^
Br1	0.0551 (5)	0.0581 (5)	0.0607 (5)	0.0174 (3)	0.0226 (4)	−0.0069 (3)
C10	0.038 (3)	0.033 (3)	0.035 (3)	0.004 (2)	0.014 (2)	0.007 (2)
N1	0.047 (3)	0.040 (2)	0.052 (3)	0.008 (2)	0.029 (3)	0.0010 (19)
C5	0.033 (3)	0.024 (2)	0.031 (2)	0.0024 (17)	0.010 (2)	0.0062 (17)
C2	0.039 (3)	0.034 (3)	0.029 (2)	−0.006 (2)	0.008 (2)	0.0037 (19)
C9	0.062 (4)	0.035 (3)	0.038 (3)	−0.005 (2)	0.014 (3)	−0.004 (2)
C6	0.033 (3)	0.034 (3)	0.036 (3)	0.003 (2)	0.015 (2)	0.011 (2)
C8	0.044 (4)	0.061 (4)	0.043 (3)	−0.012 (3)	−0.004 (3)	0.007 (3)
C1	0.060 (4)	0.036 (3)	0.032 (3)	−0.005 (2)	0.020 (3)	−0.002 (2)
C3	0.045 (3)	0.052 (3)	0.031 (3)	−0.009 (3)	0.002 (2)	0.004 (2)
C4	0.035 (3)	0.050 (3)	0.046 (3)	−0.001 (2)	0.007 (3)	0.001 (2)
N2	0.045 (3)	0.070 (4)	0.069 (4)	−0.009 (3)	0.017 (3)	0.004 (3)
C7	0.039 (3)	0.051 (4)	0.055 (4)	0.000 (2)	0.008 (3)	0.013 (3)

### Geometric parameters (Å, °)

**Table:**

Br1—C10	1.904 (5)	C9—H9A	0.9300
C10—C9	1.365 (8)	C6—C7	1.371 (7)
C10—C5	1.397 (6)	C8—C7	1.379 (9)
N1—C6	1.355 (6)	C8—H8A	0.9300
N1—C1	1.353 (8)	C1—H1B	0.9300
N1—H1A	0.8600	C3—C4	1.454 (7)
C5—C2	1.420 (7)	C3—H3A	0.9700
C5—C6	1.425 (6)	C3—H3B	0.9700
C2—C1	1.374 (7)	C4—N2	1.121 (7)
C2—C3	1.509 (7)	C7—H7A	0.9300
C9—C8	1.389 (9)		
			
C9—C10—C5	120.5 (5)	C7—C6—C5	123.7 (5)
C9—C10—Br1	118.5 (4)	C7—C8—C9	120.1 (6)
C5—C10—Br1	121.0 (4)	C7—C8—H8A	119.9
C6—N1—C1	110.0 (4)	C9—C8—H8A	119.9
C6—N1—H1A	125.0	C2—C1—N1	110.1 (5)
C1—N1—H1A	125.0	C2—C1—H1B	125.0
C10—C5—C2	136.8 (5)	N1—C1—H1B	125.0
C10—C5—C6	116.0 (5)	C4—C3—C2	112.6 (4)
C2—C5—C6	107.1 (4)	C4—C3—H3A	109.1
C1—C2—C5	106.0 (4)	C2—C3—H3A	109.1
C1—C2—C3	124.3 (5)	C4—C3—H3B	109.1
C5—C2—C3	129.7 (4)	C2—C3—H3B	109.1
C10—C9—C8	121.8 (5)	H3A—C3—H3B	107.8
C10—C9—H9A	119.1	N2—C4—C3	178.9 (6)
C8—C9—H9A	119.1	C6—C7—C8	117.8 (5)
N1—C6—C7	129.5 (5)	C6—C7—H7A	121.1
N1—C6—C5	106.8 (5)	C8—C7—H7A	121.1

### Hydrogen-bond geometry (Å, °)

**Table:**

*D*—H···*A*	*D*—H	H···*A*	*D*···*A*	*D*—H···*A*
N1—H1A···N2^i^	0.86	2.45	3.218 (7)	148.

Symmetry codes: (i) *x*+1, *y*, *z*.
